# Interaction of genetic risk score (GRS) and Plant-Based diet on atherogenic factors and body fat distribution indices among women with overweight and obesity: a cross-sectional study

**DOI:** 10.1038/s41598-025-09726-0

**Published:** 2025-07-08

**Authors:** Mahya Mehri Hajmir, Atieh Mirzababaei, Faezeh Abaj, Yasaman Aali, Mahsa Samadi, Khadijeh Mirzaei

**Affiliations:** 1 Department of Exercise and Nutrition Sciences, The George Washington University, Washington, D.C USA; 2 Chronic Diseases Research Center, Endocrinology and Metabolism Population Sciences Institute, Tehran University of Medical Sciences, Tehran, Iran; 3 Department of Community Nutrition, School of Nutritional Sciences and Dietetics, Tehran University of Medical Sciences (TUMS), Tehran, Iran; 4 Department of Nutrition, Dietetics and Food, School of Clinical Sciences at Monash Health, Monash University, Clayton, Australia; 5 Department of Nutrition, Faculty of Medicine, Mashhad University of Medical Sciences, Mashhad, Iran; 6 Department of Community Nutrition, School of Nutritional Sciences and Dietetics, Tehran University of Medical Science (TUMS), Tehran University of Medical Sciences (TUMS), P.O. Box:14155-6117, Tehran, Iran; 7 Nutrition & Dietetics, Department of Community Nutrition, School of Nutritional Sciences and Dietetics, in Tehran University of Medical Science (TUMS), Tehran University of Medical Sciences (TUMS), P.O. Box:14155-6117, Tehran, Iran

**Keywords:** A body shape index, Body roundness index, Lipid accumulation product, Body adiposity indices, Atherogenic index of plasma, Plant dietary index, Genetics, Biomarkers, Diseases, Risk factors

## Abstract

The association between plant-based diets, obesity, cardiovascular disease (CVD), and genetic predisposition is still not fully understood. This study explored how plant-based diets interact with genetic susceptibility to atherosclerosis and body fat in 377 Iranian women aged 18 to 48 who were overweight or obese. Using a validated 147-item food frequency questionnaire (FFQ), we established three plant-based diet indices: the Plant-Based Diet Index (PDI), the Healthy Plant-Based Diet Index (hPDI), and the Unhealthy Plant-Based Diet Index (uPDI). We calculated a Genetic Risk Score (GRS) based on three body mass index (BMI)-related single nucleotide polymorphisms (SNPs) and analyzed its interaction with the PDI. Results showed that tertile 2 of the PDI had a significant negative interaction with moderate and high-risk alleles on key atherogenic factors, including the atherogenic index of plasma (AIP), triglyceride glucose (TyG), lipid accumulation product (LAP), and visceral adiposity index (VAI) (*P* < 0.05). A borderline negative interaction between tertile 2 of the hPDI and moderate risk alleles regarding the Body Shape Index (ABSI) was also observed (*P* = 0.05). Conversely, tertile 2 of the uPDI had a significant positive interaction with moderate risk alleles related to both the Castelli Risk Index I (CRI.I) and II (CRI.II) (*P* = 0.03). This study suggests that interactions between genetic susceptibility and plant-based diet indices are linked to atherogenic factors and body composition.

## Introduction

Over the last few decades, obesity has become a global public health concern, affecting developed and developing countries^1^. According to a systematic review and meta-analysis in 2019, the prevalence of obesity among Iranian older adults was reported at 21.4%^2^, and it is universally more common in women^3^. It has been revealed that obesity, CVD, dyslipidemia, and metabolic abnormalities have strong associations^4^. Indeed, obesity and hyperlipidemia are recognized as the most critical risk factors for CVD^5,6^. In this regard, several obesity- or atherogenic-related predictive indices have been identified, which might predispose a person to obesity or cardiovascular diseases. ABSI and body roundness index (BRI), based on height, weight, and waist circumference (WC), have recently been proposed as new anthropometric indices. ABSI has been shown to be associated with all-cause mortality^7,8^, and BRI provides comprehensive identification of visceral adiposity tissue and body fat percentage^9^. LAP, focused on a combination of WC and triglyceride (TG), might be an accurate marker of central obesity^10^. The Body Adiposity Index (BAI) is a novel indicator of body fat percentage and has been suggested as a better predictor of health outcomes than BMI^11^. Another novel biomarker developed as a robust biomarker to predict atherosclerosis and CVD events and closely related to abdominal obesity is the AIP^12–18^. The results of a previous study have suggested that a higher AIP value is associated with a higher risk of chronic diseases^19^. Moreover, constructed indices such as CRI-I, CRI-II, and TyG index, based on lipoprotein cholesterol concentrations, are considered better predictors of atherosclerosis and CVD events^20–23^.

Among environmental factors, different dietary patterns have life-long effects on CVD and other metabolic-related risk factors^24^. Plant-based diet indices reflect the difference between plant-derived foods and their association with the risk of disease as graded scoring systems. These indices have been developed in three categories as follows: a plant-based diet index (PDI) which illustrates the whole consumption of plant food with a lower intake of animal food, a healthy plant-based diet index (hPDI), and an unhealthy plant-based diet index (uPDI)^25^. Recent studies reported that adherence to hPDI could decrease the risk of chronic diseases and improve CVDs, while diets focused on uPDI resulted in a higher risk of chronic diseases^25–29^.

The World Health Organization (WHO) identifies genetic susceptibility as a risk factor for the onset and development of CVD^30^. Gene-diet interactions are crucial for understanding individual differences in obesity and cardiometabolic risk, as they reveal how dietary factors can influence genetic predispositions. Research by Qi et al.^31^ demonstrates that genetic variants related to obesity significantly affect the body’s response to low-calorie weight-loss diets, impacting adiposity and insulin resistance. Genetic predisposition plays a significant role in determining metabolic health; however, emerging evidence suggests that plant-based diets may mitigate these risks through beneficial gene–diet interactions^31,32^. GRS is calculated by adding genetic risk alleles for each single nucleotide polymorphism (SNP)^33^, which could provide a better understanding in terms of trait variability of an individual and improve genetic risk prediction affecting the variables tested in this context compared to a single variant method^34^. Caveolin-1 (CAV-1), abundant in adipocytes^35^, was previously reported to be associated with obesity, dyslipidemia, and atherosclerosis^36–39^. Furthermore, the risk allele C for melanocortin 4 receptor (MC4R) rs17782313 is considered a key factor in developing obesity and increased cardiovascular risk factors^40–44^. Cryptochromes 1 (Cry 1) have also been shown to play critical roles in metabolism regulation, obesity, and elevated cardiometabolic traits^45–47^. Many studies have reported the contribution of healthy dietary patterns and PDI in reducing genetic risk factors of obesity and CVD^48,49^. However, no study has examined the association between PDI and genetic factors and their impact on atherogenic factors and body fat distribution Indices among women. Therefore, this study aimed to evaluate the interaction between Genetic Risk Score (GRS) and plant-based diet indices (PDI, hPDI, and uPDI) on atherogenic factors and body fat distribution indices among women with overweight and obesity.

## Methods and materials

### Study population

This cross-sectional study involved 377 women aged 18–48 from Tehran, Iran, with a BMI of 25 to 40 kg/m², who were referred to health centers associated with the Tehran University of Medical Sciences (TUMS) from 2017 to 2019. A multistage cluster random sampling method was used to select specific regions within the city to ensure a representative sample. In total, 20 clusters were identified from which the participants were recruited. Subjects with the following conditions were excluded in this study: pregnancy, lactation, menopause, a history of diseases including type I and type II diabetes, non-alcoholic fatty liver disease (NAFLD), thyroid illness, kidney or liver diseases, polycystic ovary syndrome (PCOS), malignancies, and stroke. **Exclusion criteria included using supplements or medications**,** participating in a weight loss program**,** or having total calorie intake outside the 800–4200 kcal/day range.** Before enrolling, all participants completed the informed consent form, which was reviewed and approved by the Ethics Committee of the TUMS (NO: IR. TUMS.VCR.REC.1398.142). This study was performed according to relevant guidelines and regulations.

### General, anthropometric, and physical activity assessments

Participants provided demographic information through a self-reported sociodemographic questionnaire, which included details on age, marital status (single or married), education level (under diploma, diploma, or university), occupation (employed, unemployed, or student), current smoking status (yes or no), and supplement use (yes or no). Additionally, an individual’s economic status was assessed using a 9-question self-reported questionnaire. Participants who received scores of 0 to 3 were classified as having a poor economic level. Those with scores ranging from 4 to 7 were deemed to have a moderate economic level, while individuals scoring 8 to 9 were considered to be in a good economic level^50^. Weight measurement was recorded with light clothes using a digital scale (Seca, Germany) with a precision of 0.1 cm and 0.1 kg. Also, height was recorded using a calibrated height gauge (in the standing position without shoes). BMI was calculated by dividing weight (kg) by the square of height (m^2^). Waist circumference was assessed using tape, ensuring no pressure was applied to the body during measurement. This measurement was taken after a natural exhalation, focusing on the narrowest part of the waist, with an accuracy of 0.5 cm. Similarly, hip circumference was measured at the most prominent point without exerting pressure, ensuring the same level of precision. The waist-to-hip ratio was calculated by dividing the waist circumference by the hip circumference. Overweight and obesity were defined as BMI of 25–29.9 kg/m^2^ and a BMI of 30–40 kg/m^2^, respectively^51^.

Body composition was assessed using bioelectrical impedance analysis device (BIA 770 (South Korea)), following manufacturer guidelines^52^. In addition, physical activity (PA) was evaluated using the validated self-report International Physical Activity Questionnaire (IPAQ) short form^53^. The IPAQ, developed in 1998 by a panel of experts, serves as a global standard for monitoring physical activity. It includes two versions: a long-form with 31 items and a short form with 9 items, both of which have established validity and reliability, as confirmed by a study conducted by Craig et al. in 2003^54^. A 9-item questionnaire assessing physical activity over the past week, including walking, moderate, and vigorous activities, as well as sitting time. Each activity type is assigned a standard MET value: 3.3 for walking, 4.0 for moderate, and 8.0 for vigorous activity. MET minutes per week are calculated by multiplying the MET value by the minutes and days per week for each activity. Total physical activity is obtained by summing the MET-min/week across all activity types^55–57^.

### Laboratory tests

All blood samples were obtained after 12–14 h of fasting, centrifuged and stored at −80 °C at the Nutrition and Biochemistry Laboratory of the School of Nutritional and Dietetics at TUMS. Fasting blood sugar (FBS) was measured using the Glucose Oxidase Phenol 4-Aminoantipyrine Peroxidase (GOD/PAP) method. A radioimmunoassay was used to measure serum insulin values. All lipid biomarkers, including total cholesterol (T-Chol, mg/dl), low-density lipoprotein (LDL, mg/dl), high-density lipoprotein (HDL, mg/dl), and triglycerides (TG, mg/dl), were measured using enzymatic methods (Pars Azmun Co., Tehran, Iran).

### Atherogenic index of plasma (AIP) and lipid ratio assessment

The atherogenic index of plasma (AIP) was calculated from the logarithmic ratio of TG to HDL-C. Lipid ratios were calculated as follows: CRI - I = TC/HDL-C, CRI – II = LDL-C/HDL-C^58^. TyG was estimated using the formula: Ln [fasting triglycerides (mg/dl) × FPG (mg/dl)/2]^59,60^ and LAP = (WC − 58) × TG^61^.

### *A body shape index (ABSI)*,* body roundness index (BRI)*,* and Body adiposity index (BAI) definitions*.

*A body shape index (ABSI)*,* body roundness index (BRI)*,* and Body adiposity index (BAI) definitions*.

The ABSI, BRI, and BAI were calculated using the following articles^7,9,11^. ABSI was derived from WC and adjusted for height and weight.

ABSI = WC/BMI^2/3^ Height^1/2^.

BRI was calculated as follows:

BRI = 364.2–365.5 Eccentricity.

Eccentricity calculates the degree of circularity of an ellipse, which ranges between 0 and 1, with 0 characterizing a perfect circle and 1, a vertical line.

BAI was calculated using the formula: BAI = Hip/Height ^ X.

In this formula, the hip reflects hip circumference (in cm), height is measured in meters, and where X is a unitless power term.

### Dietary assessments and plant-based dietary pattern

Dietary intake was assessed using a validated 147-item semi-quantitative FFQ with previously approved validity and reliability^62^. In the presence of expert dietitians, subjects were asked to report their consumption frequency during the past year based on their usual diet, which was converted to grams per day. Total energy and dietary nutrients were analyzed using the Iranian Food Composition Table (FCT) and N4 software.

Three plant-based dietary intakes, including overall PDI, hPDI, and uPDI, were calculated using the method proposed by Satija *et*al^63^.. In brief, all food intakes were divided into 18 groups, which were animal foods (dairy, animal fat, egg, fish and seafood, meat, miscellaneous animal-based foods), healthy (whole grains, fruits, vegetables, nuts, legumes, vegetable oils, tea, and coffee), and unhealthy plant-based diets (fruit juices, sugar-sweetened beverages, refined grains, potatoes, sweets, and desserts)^25,64^. Eighteen energy-adjusted food groups were divided into quintiles, with scores ranging from 1 to 5 assigned based on positive or reverse scoring criteria. Focusing on positive scores, a score of 5 was given to the highest quintiles, and a score of 1 was assigned to the lowest quintiles, whereas this pattern was inversed for reverse scores. For PDI, both healthy and unhealthy plant-based foods received positive scores. For hPDI and uPDI, only healthy and unhealthy plant foods received positive scores, respectively. Animal food groups were given reverse ratings in all three indices. Finally, the observed scores for each plant-based diet index ranged from 18 to 90, and a higher total score was associated with higher adherence to that diet index^25^.

### Genotyping and GRS

DNA was extracted using the salting-out method^65^, and its integrity was assessed using a 1% agarose gel. DNA concentration was assessed by a nanodrop 8000 Spectrophotometer (Thermo Scientific, Waltham, MA, USA). Genotyping of the SNPs was performed using the PCR-allele technique with the TaqMan Open Array (Life Technologies Corporation, Carlsbad, CA, USA)^66^. For CAV-1 (rs3807992), the forward primer is 3′AGTATTGACCTGATTTGCCATG 5′, and the reverse primer is 5′ GTCTTCTGGAAAAAGCACATGA 3′. The fragments containing three genotypes (GG, GA, and AA) were distinguished. The MC4R gene primer was selected based on a previous study^67^. The forward and reverse primer of MC4R (rs17782313) are 5- AAGTTCTACCTACCATGTTCTTGG-3 and 5-TTCCCCCTGAAGCTTTTCTTGTCATTTTGAT-3, respectively. The fragments containing three genotypes (CC, CT, and TT) were distinguished. We used PCR with the following primers for Cry1 (rs2287161): the forward primer is 5′-GGAACAGTGATTGGCTCTATCT − 3′, and the reverse primer is 5′-GGTCCTCGGTCTCAAGAAG-3′. The fragments containing three genotypes (CC, GC, and GG) were distinguished.

The Genetic Risk Score (GRS) was created by summing the risk alleles of three single nucleotide polymorphisms [CAV-1 (rs3807992), Cry-1 (rs2287161), and MC4R (rs17782313)], which have been associated with obesity-related traits in large-scale genome-wide association studies (GWAS)^68–72^. Genotypes were coded as 0, 1, or 2 based on the number of risk alleles associated with higher BMI for each SNP. An unweighted GRS was calculated using the risk alleles of the three SNPs. The GRS ranges from 0 to 6, with higher scores indicating a greater genetic predisposition to higher BMI or body weight^73^.

### Statistical analysis

The sample size of 377 participants was estimated based on Peduzzi’s method for logistic regression, which recommends a minimum of 10 participants per predictor variable. Given the planned analysis of three plant-based diet indices, genetic risk categories, and their interactions (resulting in approximately 12 analytic conditions), this sample size was considered sufficient to detect moderate interaction effects with acceptable statistical power.

The Kolmogorov-Smirnov test was used to assess the normality of the data distribution. The Hardy-Weinberg equilibrium and categorical variable comparisons were assessed with the chi-square test. Descriptive analysis was used to evaluate demographic characteristics, with continuous variables reported as mean ± SD and categorical variables as N (%). Analysis of variance (ANOVA) was used to compare anthropometric measurements and metabolic profiles between subjects, while analysis of covariance (ANCOVA) was used to adjust for confounding variables. Analyses were adjusted for age, BMI, physical activity, and total energy intake. Logistic regression was used to account for confounding variables in categorical data. The generalized linear model (GLM) was used to evaluate the interactions between GRS and PDI. Data analysis was performed using SPSS (version 25; SPSS Inc., IL). One-sided P-values were used; a P-value < 0.05 was considered statistically significant, and a P-value < 0.1 was considered significant for interactions.

## Results

### Study population characteristics based on GRS

The present study included 377 Iranian women. The characteristics of individuals are presented in Table 1. There was a significant difference between GRS with body weight (P-value = 0.03), BMI (P-value = 0.02), WC (P-value = 0.03), and WHR (P-value = 0.03) in the crude model. After adjustment for confounders (BMI, age, total energy intake, physical activity), there was a significant mean difference for the body weight (P-value = 0.04), BMI (P-value = 0.01), WC (P-value = 0.03), WHR (P-value = 0.01), BRI (P-value = 0.02), ABSI (P-value = 0.03), and LAP (P-value = 0.02) in participants.

**Table 1:** Characteristics of the study population among participants based on GRS.

Quantitative variables	No risk (< 3 risk allele) (*N* = 123)	Moderate risk (3&4 risk allele) (*N* = 171)	High risk (≥ 5 risk allele) (*N* = 42)	*P*-value	*P*-value*
Age (year)	35.97 ± 8.99	36.39 ± 8.01	36.32 ± 8.51	0.94	0.81
Anthropometric indices					
Body weight (kg)	79.38 ± 9.59	78.002 ± 11.03	83.46 ± 10.63	0.03	0.04
Height (cm)	162.37 ± 5.29	160.78 ± 6.12	160.93 ± 4.35	0.11	0.15
BMI (kg/m^2^)	30.11 ± 3.40	30.17 ± 3.70	31.30 ± 3.33	0.02	0. 01
WC (cm)	97.57 ± 8.66	96.60 ± 8.90	101.14 ± 8.96	0.03	0.03
WHR (cm)	0.92 ± 0.05	0.92 ± 0.04	0.95 ± 0.05	0.03	0.01
Body composition					
BRI	5.47 ± 1.18	5.58 ± 1.25	6.09 ± 1.21	0.08	0.02
ABSI	0.79 ± 0.02	0.78 ± 0.02	0.79 ± 0.03	0.33	0.03
LAP	54.49 ± 34.08	49.52 ± 28.93	64.66 ± 40.31	0.09	0.02
VAI	290.63 ± 184.80	286.32.196.71	379.24 ± 302.10	0.11	0.09
Biochemical parameter					
TC (mg/dl)	187.26 ± 33.37	183.01 ± 37.01	176.84 ± 37.80	0.41	0.73
TG (mg/dl)	120.67 ± 58.05	110.69 ± 55.20	133.72 ± 75.79	0.17	0.06
LDL-c (mg/dl)	97.37 ± 22.15	95.13 ± 25.06	88.32 ± 27.44	0.26	0.62
HDL-c (mg/dl)	47.38 ± 9.58	47.14 ± 11.61	45.92 ± 12.32	0.84	0.96
FBS (mg/dl)	87.17 ± 9.08	86.62 ± 10.14	88.56 ± 9.64	0.66	0.60
Atherogenic index					
AIP	0.36 ± 0.22	0.34 ± 0.25	0.42 ± 0.28	0.29	0.19
CRI.1	4.04 ± 0.94	4.14 ± 1.80	4.16 ± 1.38	0.88	0.96
CRI.II	2.08 ± 0.50	2.09 ± 0.61	2.02 ± 0.64	0.83	0.92
TyG	1.39 ± 0.71	1.28 ± 0.59	1.48 ± 0.82	0.31	0.09
Qualitative variables	N (%)			P-value	P-value*
Marital situation					
Single	29 (49.2)	25 (42.4)	5 (8.5)	0.16	0.18
Married	69 (35.9)	96 (50)	27 (14.1)		
Education					
Less diploma	15 (41.7)	14 (38.9)	7 (19.4)	0.30	0.32
Diploma	34 (35.1)	48 (49.5)	15 (15.5)		
Bachelor or higher	49 (42.2)	57 (49.1)	10 (8.6)		
Job					
Employed	45 (46.9)	40 (41.7)	11 (11.5)	0.16	0.20
Unemployed	52 (34.7)	77 (51.3)	21 (14)		
Economic status					
Poor	19 (34.5)	27 (49.1)	9 (16.4)	0.77	0.84
Moderate	46 (39.3)	56 (47.9)	15 (12.8)		
Good	28 (43.8)	30 (46.9)	6 (9.4)		

BMI: body mass index, WC: waist circumference, WHR: waist hip ratio, AIP: atherogenic index of plasma, AC: atherogenic coefficient, CRI−I: Castelli risk index 1, CRI−II: Castelli risk index II, TyG: Triglyceride−glucose index, BRI: body roundness index, ABSI: a body shape index, LAP: lipid accumulation product, VAI: visceral adiposity index, TC: total cholesterol, TG: triglyceride, LDL−c: low−density lipoprotein, HDL−c: high−density lipoprotein, FBS: fasting blood sugar, CAV−1: Caveolin, CRY: Cryptochrome Circadian Regulator, MC4R: Melanocortin 4 Receptor.

Quantitative variables as means±SD obtained from the ANOVA test.

Qualitative variables N (%) obtained from the chi−square analysis.

P−value*obtained from ANCOVA test. P−values < 0.05 were considered significant.

P−value*for adjustment model, based on age, total energy intake, BMI, and physical activity.

### Study population characteristics according to tertile of PDI, hPDI, uPDI

The first tertile of adherence to the dietary patterns (PDI, hPDI, and uPDI) was used as the reference group for comparison. There was no significant difference between the tertiles of PDI with characteristics of the study population in the crude and adjusted model (P-value > 0.05), except education (P-value = 0.02) and job status (P-value = 0.002) in the crude model and education (P-value = 0.01) and job status (P-value = 0.001) in the adjusted model in Table 2. The research findings indicated that adherence to the hPDI increased with the age of the participants, showing a statistically significant difference (*p* = 0.002). This trend persisted even after adjusting for potential confounding factors (*p* = 0.003). Furthermore, upon accounting for these confounders, there was a borderline significant difference for TyG across the tertiles of hPDI (P-value = 0.05) and a significant difference for TG (P-value = 0.04) in the adjusted model. Also, a significant difference was observed for economic status (P-value < 0.001) in participants in crude and adjusted models. The crude model showed a significant difference for BMI across the tertiles of the uPDI (P-value = 0.02) and BRI (P-value = 0.03). The observed relation remained significant even after adjustment for confounding variables for BMI (P-value = 0.03) and BRI (P-value = 0.04).

**Table 2:** Characteristics of study population in among tertiles of PDI, hPDI, uPDI.

Variables	PDI					hPDI					uPDI				
	T1 (*N* = 111) (< 51 gr)	T2 (*N* = 91) (51–57 gr)	T3 (*N* = 88) (≥ 57)	P-value	P-value*	T1 (*N* = 97) (< 51 gr)	T2 (*N* = 106) (51–57 gr)	T3 (*N* = 87) ((≥ 57)	P-value	P-value*	T1 (*N* = 104) (< 45 gr)	T2 (*N* = 100) (45–51 gr)	T3 (*N* = 86) (≥ 51)	P-value	P-value*
Age (year)	36.29 ± 8.93	36.00 ± 8.23	36.57 ± 8.11	0.92	0.91	33.46 ± 7.94	37.02 ± 8.77	38.07 ± 7.98	0.002	0.003	37.36 ± 8.62	35.87 ± 8.44	35.40 ± 8.22	0.31	0.22
Anthropometric indices and															
Body weight (kg)	79.09 ± 10.51	79.82 ± 10.25	80.08 ± 11.15	0.79	0.82	79.21 ± 10.26	80.08 ± 10.83	79.48 ± 10.75	0.84	0.91	79.04 ± 10.11	81.75 ± 11.30	77.89 ± 10.05	0.04	0.03
Height (cm)	160.72 ± 5.90	162.30 ± 6.26	160.69 ± 5.38	0.11	0.18	162.21 ± 6.12	160.88 ± 5.48	160.54 ± 6.07	0.13	0.23	161.58 ± 5.36	160.43 ± 5.74	161.64 ± 6.61	0.28	0.31
BMI (kg/m^2^)	30.18 ± 3.83	30.13 ± 3.32	31.04 ± 3.84	0.25	0.24	29.60 ± 3.27	30.77 ± 4.07	30.80 ± 3.55	0.07	0.08	30.25 ± 3.48	31.28 ± 3.95	29.62 ± 3.48	0.02	0.03
WC (cm)	96.60 ± 8.73	98.24 ± 9.35	98.51 ± 9.57	0.34	0.76	96.95 ± 8.50	97.91 ± 9.69	98.07 ± 9.29	0.72	0.45	97.35 ± 9.01	99.47 ± 9.44	95.94 ± 8.82	0.06	0.07
WHR (cm)	0.92 ± 0.04	0.93 ± 0.05	0.92 ± 0.04	0.25	0.53	0.93 ± 0.04	0.92 ± 0.04	0.93 ± 0.05	0.68	0.77	0.92 ± 0.05	0.93 ± 0.04	0.92 ± 0.04	0.24	0.26
Body composition															
BRI	5.55 ± 1.28	5.59 ± 1.25	5.78 ± 1.31	0.50	0.42	5.47 ± 1.14	5.72 ± 1.44	5.68 ± 1.22	0.45	0.52	5.56 ± 1.20	5.91 ± 1.34	5.39 ± 1.27	0.03	0.04
ABSI	0.78 ± 0.02	0.79 ± 0.02	0.78 ± 0.02	0.16	0.29	0.79 ± 0.02	0.78 ± 0.02	0.78 ± 0.02	0.09	0.19	0.79 ± 0.02	0.79 ± 0.02	0.78 ± 0.02	0.93	0.88
LAP	56.47 ± 37.76	49.66 ± 28.56	53.28 ± 29.52	0.42	0.45	53.29 ± 29.07	49.97 ± 30.58	57.50 ± 37.96	0.35	0.27	50.86 ± 28.71	58.33 ± 37.69	50.91 ± 31.08	0.26	0.30
VAI	318.20 ± 230.52	270.06 ± 184.37	301.60 ± 197.44	0.34	0.37	305.21 ± 203.12	269.42 ± 165.55	325.41 ± 249.12	0.22	0.20	274.08 ± 173.59	313.02 ± 210.49	312.81 ± 241.89	0.39	0.33
Biochemical parameter															
TC (mg/dl)	185.21 ± 39.06	182.70 ± 33.47	185.83 ± 35.04	0.85	0.93	183.97 ± 38.22	182.18 ± 37.71	188.07 ± 32.33	0.56	0.45	184.97 ± 34.89	185.51 ± 37.15	183.24 ± 36.97	0.92	0.90
TG (mg/dl)	122.54 ± 64.54	109.02 ± 52.13	120.54 ± 59.66	0.31	0.27	123.67 ± 64.98	106.90 ± 51.73	124.65 ± 61.46	0.09	0.04	112.92 ± 52.52	122.66 ± 65.90	118.75 ± 60.74	0.55	0.60
LDL-c (mg/dl)	96.20 ± 24.87	93.85 ± 23.96	94.62 ± 23.86	0.81	0.87	91.06 ± 24.31	96.37 ± 24.90	97.36 ± 23.18	0.21	0.53	96.86 ± 24.02	96.17 ± 25.24	91.27 ± 23.12	0.30	0.21
HDL-c (mg/dl)	47.32 ± 10.81	47.50 ± 10.83	46.10 ± 10.50	0.68	0.74	46.13 ± 10.009	47.95 ± 11.12	46.81 ± 10.90	0.54	0.67	48.43 ± 11.21	46.57 ± 9.69	45.69 ± 11.11	0.25	0.07
FBS (mg/dl)	86.81 ± 10.07	87.16 ± 8.17	88.36 ± 10.29	0.56	0.62	87.18 ± 8.88	87.97 ± 10.80	86.94 ± 8.94	0.77	0.57	86.41 ± 7.86	87.89 ± 10.42	88.04 ± 10.61	0.48	0.37
Atherogenic index															
AIP	0.38 ± 0.25	0.32 ± 0.23	0.36 ± 0.23	0.34	0.34	0.37 ± 0.26	0.32 ± 0.21	0.38 ± 0.25	0.28	0.22	0.34 ± 0.21	0.36 ± 0.25	0.37 ± 0.27	0.75	0.53
CRI.1	4.16 ± 1.84	3.95 ± 0.95	4.12 ± 1.23	0.65	0.70	4.24 ± 2.16	3.94 ± 1.01	4.10 ± 0.91	0.43	0.15	3.93 ± 0.83	4.06 ± 1.06	4.31 ± 2.23	0.27	0.22
CRI.II	2.09 ± 0.55	2.03 ± 0.56	2.08 ± 0.59	0.75	0.84	2.003 ± 0.56	2.09 ± 0.63	2.10 ± 0.49	0.48	0.80	2.05 ± 0.50	2.10 ± 0.60	2.06 ± 0.62	0.86	0.73
TyG	1.42 ± 0.71	1.25 ± 0.62	1.31 ± 0.60	0.26	0.30	1.37 ± 0.68	1.21 ± 0.50	1.43 ± 0.75	0.08	0.05	1.32 ± 0.63	1.37 ± 0.70	1.32 ± 0.62	0.86	0.77
Qualitative variables	N (%)			P-value	P-value*	N (%)			P-value	P-value*	N (%)			P-value	P-value*
Frequency															
Low-risk allele	31 (31.6)	32 (32.7)	35 (35.7)	0.58	0.42	35 (35.7)	36 (36.7)	27 (26.6)	0.91	0.88	40 (40.8)	31 (31.6)	27 (27.6)	0.75	0.91
Moderate risk allele	49 (42.2)	33 (28.4)	34 (29.3)			36 (31)	42 (36.2)	38 (32.8)			43 (37.1)	35 (30.2)	38 (32.8)		
High risk allele	10 (33.3)	9 (30)	11 (36.7)			9 (30)	11 (36.7)	10 (33.3)			11 (36.7)	12 (40)	7 (23.3)		
Marital situation															
Single	26 (39.4)	16 (24.2)	24 (36.4)	0.29	0.33	27 (40.9)	22 (33.3)	17 (25.8)	0.33	0.28	22 (33.3)	21 (31.8)	23 (34.8)	0.57	0.63
Married	85 (37.9)	75 (33.5)	64 (28.6)			70 (31.3)	84 (37.5)	70 (31.3)			82 (36.6)	79 (35.3)	63 (28.1)		
Education															
Less diploma	10 (23.3)	13 (30.2)	20 (46.5)	0.02	0.01	10 (23.3)	12 (27.9)	21 (48.8)	0.06	0.07	10 (23.3)	16 (37.2)	17 (39.5)	0.10	0.23
Diploma	39 (35.1)	39 (35.1)	33 (29.7)			36 (32.4)	44 (39.6)	31 (27.9)			36 (32.4)	43 (38.7)	32 (28.8)		
Bachelor or higher	62 (46.3)	38 (25.4)	34 (25.4)			50 (37.3)	49 (36.6)	35 (26.1)			58 (43.3)	39 (29.1)	37 (27.6)		
Job															
Employed	56 (50)	31 (27.7)	25 (22.3)	0.002	0.001	34 (30.4)	45 (40.2)	33 (29.5)	0.46	0.51	40 (35.7)	36 (32.1)	36 (32.1)	0.80	0.83
Unemployed	51 (29.7)	59 (34.3)	62 (36)			61 (35.5)	57 (33.1)	54 (31.4)			61 (35.5)	61 (35.5)	50 (29.1)		
Economic status															
Poor	21 (31.3)	25 (37.3)	21 (31.3)	0.58	0.64	15 (22.4)	17 (25.4)	35 (52.2)	< 0.001	< 0.001	27 (40.3)	23 (34.3)	17 (25.4)	0.21	0.22
Moderate	55 (39.9)	40 (29)	43 (31.2)			50 (36.2)	58 (42)	30 (21.7)			45 (32.6)	42 (30.4)	51 (37)		
Good	31 (43.7)	21 (29.6)	19 (26.8)			27 (38)	25 (35.2)	19 (26.8)			29 (40.8)	26 (36.6)	16 (22.5)		

BMI: body mass index, WC: waist circumference, WHR: waist−hip ratio, AIP: atherogenic index of plasma, AC: atherogenic coefficient, CRI−I: Castelli risk index 1, CRI−II: Castelli risk index II, TyG: Triglyceride−glucose index, BRI: body roundness index, ABSI: a body shape index, LAP: lipid accumulation product, VAI: visceral adiposity index, TC: total cholesterol, TG: triglyceride, LDL−c: low−density lipoprotein, HDL−c: high−density lipoprotein, FBS: fasting blood sugar.

Quantitative variables as means±SD were obtained from the ANOVA test.

Qualitative variables N (%) were obtained from the chi−square analysis.

P−value*obtained from ANCOVA test. P−values < 0.05 were considered significant.

P−value*for adjustment model, based on BMI, age, total energy intake, physical activity.

### Dietary intakes according to GRS in participants

The crude model showed a significant difference between GRS and glucose (P-value = 0.03) in Table 3. Also, there was a borderline significance between GRS and vitamin B9 (P-value = 0.05) and fructose (P-value = 0.05). After adjustment for confounding variables (BMI, age, total energy intake, physical activity), there was a significant difference between GRS and Vitamin C (P-value = 0.03) and glucose (P-value = 0.02).

**Table 3:** Dietary intakes according to GRS in participants.

Variables	No risk (*N* = 123)	Moderate risk (*N* = 171)	High risk (*N* = 42)	*P*-value	*P*-value*
Macronutrients					
Energy (Kcal)	2745.61 ± 729.83	2561.11 ± 705.13	2632.58 ± 781.46	0.18	0.23
Carbohydrates (gr/day)	394.97 ± 122.13	366.21 ± 111.29	366.74 ± 127.05	0.17	0.51
Protein (gr/day)	93.31 ± 28.70	86.85 ± 26.84	90.23 ± 34.62	0.26	0.92
Total fat (gr/day)	97.60 ± 28.77	91.59 ± 31.57	96.66 ± 33.69	0.33	0.80
Subgroup types of fat					
CHOL (gr/day)	264.53 ± 107.65	245.32 ± 100.88	252.27 ± 115.31	0.41	0.94
Saturated fat (gr/day)	29.05 ± 11.42	27.35 ± 10.21	28.09 ± 12.21	0.52	0.85
Trans fat (gr/day)	0.0008 ± 0.002	0.0007 ± 0.001	0.001 ± 0.002	0.28	0.30
MUFA (gr/day)	32.05 ± 9.78	30.53 ± 10.75	31.99 ± 13.14	0.55	0.85
PUFA (gr/day)	20.54 ± 7.30	19.27 ± 8.31	20.80 ± 8.30	0.42	0.98
Micronutrients					
Vitamins					
B1 (mg/day)	2.18 ± 0.66	2.02 ± 0.59	2.15 ± 0.80	0.19	0.45
B2 (mg/day)	2.32 ± 0.82	2.13 ± 0.72	2.23 ± 1.02	0.23	0.44
B3 (mg/day)	26.89 ± 10.001	24.30 ± 7.52	26.77 ± 12.64	0.10	0.28
B6 (mg/day)	2.31 ± 0.71	2.12 ± 0.68	2.15 ± 0.79	0.15	0.64
B9 (mg/day)	645.44 ± 170.50	588.25 ± 171.45	624.19 ± 188.19	0.05	0.32
B12 (mg/day)	4.36 ± 2.005	4.45 ± 2.37	4.38 ± 2.86	0.95	0.35
Vitamin D (µ/day)	1.99 ± 1.78	2.09 ± 1.52	1.87 ± 1.64	0.77	0.40
Vitamin E (mg/day)	18.03 ± 8.60	16.41 ± 7.83	18.49 ± 11.26	0.28	0.66
Vitamin C (mg/day)	218.25 ± 120.82	184.05 ± 107.09	191.73 ± 203.72	0.14	0.03
Minerals					
Calcium (mg/day)	1220.89 ± 416.63	1154.14 ± 416.84	1106.32 ± 350.03	0.30	0.58
Iron (mg/day)	19.86 ± 5.95	18.07 ± 5.41	18.95 ± 6.92	0.08	0.40
Phosphor (mg/day)	1716.34 ± 524.59	1617.40 ± 507.05	1614.48 ± 491.42	0.33	0.72
Magnesium (mg/day)	485.88 ± 148.74	452.92 ± 143.96	445.74 ± 145.94	0.19	0.19
Zinc (mg/day)	13.54 ± 4.18	12.75 ± 4.05	12.97 ± 4.34	0.37	0.65
Selenium	125.76 ± 45.19	117.41 ± 40.64	119.56 ± 43.38	0.35	0.90
Other					
Total fiber (gr/day)	48.48 ± 19.67	43.51 ± 16.46	45.004 ± 22.50	0.14	0.35
Total sugar	152.22 ± 60.67	142.23 ± 59.75	126.34 ± 51.69	0.10	0.05
Glucose	22.99 ± 13.39	20.69 ± 10.94	16.71 ± 7.99	0.03	0.02
Galactose	2.77 ± 1.98	2.69 ± 1.80	2.59 ± 1.55	0.88	0.94
Fructose	27.33 ± 14.92	25.22 ± 13.31	20.54 ± 10.60	0.05	0.06
Caffeine	158.36 ± 210.20	145.54 ± 98.47	156.46 ± 137.65	0.82	0.77

CHOL: cholesterol, MUFA: monounsaturated fatty acid, PUFA: polyunsaturated fatty acid.

All data are presented as mean±SD.

P−value*for adjustment model, based on BMI, age, total energy intake, physical activity.

### Interaction between GRS and PDI with atherogenic index and body composition

The findings revealed (in Table 4) a negative significant interaction between tertile 2 of PDI, moderate risk alleles (β =−0.34, 95% CI= −0.61 to −0.07, *p* = 0.01), high-risk alleles (β =−0.38, 95% CI= −0.64 to −0.12, P-value = 0.004) and AIP compare to low-risk alleles. The observed relation remained significant even after adjustment for confounding variables. There was a negative significant interaction between tertile 2 of PDI, high-risk alleles (β =−0.63, 95% CI= −1.28 to 0.01, P-value = 0.05) and CRI.II compare to low-risk allele participants in the adjusted model.

There was a negative significant interaction between tertile 2 of PDI, moderate risk allele (β =−0.83, 95% CI= −1.57 to −0.09, P-value = 0.02), high-risk allele (β =−1.004, 95% CI= −1.71 to −0.28, P-value = 0.006) and TyG compare to low-risk allele participants. The observed relation remained significant even after adjustment for confounding variables.

There was a negative significant interaction between tertile 2 of PDI, moderate risk allele (β =−56.53, 95% CI= −97.12 to −15.94, P-value = 0.006), high-risk alleles (β =−66.72, 95% CI= −105.95 to −27.49, P-value = 0.001) and LAP compare to low-risk allele participants. After adjusting for potential confounders, there was a negative significant interaction between tertile 2 of PDI, moderate risk allele (β = −78.06, 95% CI= −120.23 to −35.90, P-value = < 0.001), high-risk alleles (β =−83.85, 95% CI= −124.45 to −43.25, P-value < 0.001) and LAP compare to low-risk allele participants.

There was a negative significant interaction between tertile 2 of PDI, moderate risk allele (β =−273.40, 95% CI= −499.34 to −47.45, P-value = 0.01), high-risk alleles (β =−311.03, 95% CI= −529.61 to −92.45, P-value = 0.005) and VAI compare to low-risk allele participants, in the crude model. There was a negative significant interaction between tertile 2 of PDI, moderate risk allele (β =−362.91, 95% CI= −611.44 to −114.38, P-value = 0.004), high-risk alleles (β =−393.001, 95% CI= −632.49 to −153.50, P-value = 0.001) and VAI compare to low-risk allele participants in adjusted model (Table 4).

### *Interaction between* GRS *and hPDI with atherogenic index and body composition*

There was a negative significant interaction between tertile 2 of hPDI, moderate risk allele (β =−0.03, 95% CI= −0.06 to −0.01, P-value = 0.008) and ABSI compared to low-risk allele participants. After adjusting for potential confounders, the observed relation remained significant (β =−0.02, 95% CI= −0.05 to 0.001, P-value = 0.05) (Table 4).

### *Interaction between* GRS *and uPDI with atherogenic index and body composition*

In the crude model, there was a positive significant interaction between tertile 2 (β = 2.11, 95% CI = 0.39 to 3.83, P-value = 0.01), tertile 3 (β = 1.90, 95% CI = 0.18 to 3.61, P-value = 0.03) of uPDI, moderate risk allele and CRI. I compare to low-risk allele participants. In the adjusted model, there was a positive significant interaction between tertile 2 (β = 2.03, 95% CI = 0.17 to 3.89, P-value = 0.03) of uPDI, moderate risk allele, and CRI. I compare to low-risk allele participants. After adjusting for potential confounder, there was a positive significant interaction between tertile 2 (β = 0.78, 95% CI = 0.07 to 1.48, P-value = 0.03) of uPDI, moderate risk allele, and CRI. II compared to low-risk allele participants.

In the adjusted model, there was a positive significant interaction between tertile 2 (β = 0.03, 95% CI = 0.009 to 0.06, P-value = 0.01) of uPDI, moderate risk allele, and ABSI. Also, there was a positive significant interaction between tertile 3 (β = 0.03, 95% CI = 0.005 to 0.06, P-value = 0.02) of uPDI, high-risk allele, and ABSI compared to low-risk allele participants (Table 4).

**Table 4:** Interaction between GSR and PDI, hPDI, and uPDI with atherogenic index and body composition in participants.

PDI										hPDI							
	GRS	T2				T3				T2				T3			
		Crude	P-value	Adjust	P-value	Crude	P-value	Adjust	P-value	Crude	P-value	Adjust	P-value	Crude	P-value	Adjust	P-value
		β (95% Cl)		β (95% Cl)		β (95% Cl)		β ± 95% Cl		β ± 95% Cl		β ± 95% Cl		β ± 95% Cl		β ± 95% Cl	
Atherogenic index																	
AIP	Moderate risk	−0.34 (−0.61,−0.07)	0.01	−0.42 (−0.71,−0.13)	0.004	−0.16± (−0.42,0.09)	0.21	−0.20± (−0.48,0.08)	0.16	0.01± (−0.25,0.28)	0.90	0.01± (−0.27,0.31)	0.90	0.10± (−0.17,0.37)	0.46	0.10± (−0.20.0.42)	0.50
	High risk	−0.38 (−0.64,−0.12)	0.004	−0.49 (−0.77,−0.21)	< 0.001	−0.15± (−0.41,0.10)	0.23	−0.23± (−0.52,0.04)	0.09	−0.03± (−0.29,0.22)	0.80	−0.02± (−0.30,0.25)	0.85	0.06± (−0.19,0.33)	0.61	0.10± (−0.20,0.40)	0.51
CRI.I	Moderate risk	−0.69 (−2.35,0.96)	0.41	−0.64 (−2.45,1.17)	0.48	−0.28± (−1.93,1.37)	0.73	−0.42± (−2.28,1.43)	0.65	−0.71± (−2.39,0.95)	0.39	−0.70± (−2.55,1.13)	0.45	−0.38± (−2.03,1.27)	0.65	−0.23± (−2.13,1.66)	0.81
	High risk	−0.20 (−1.80,1.40)	0.80	−0.20 (−1.94,1.53)	0.81	−0.25± (−1.89,1.38)	0.76	−0.63± (−2.46,1.20)	0.50	−0.18± (−1.80,1.43)	0.82	−0.003± (−1.77,1.76)	0.99	0.27± (−1.33,1.88)	0.73	0.49± (−1.33,2.32)	0.59
CRI.II	Moderate risk	−0.41 (−1.04,0.21)	0.19	−0.50 (−1.17,0.17)	0.14	−0.35± (−0.98,0.27)	0.26	−0.49± (−1.18,0.19)	0.16	−0.34± (−0.98,0.28)	0.28	−0.31± (−1.009,0.38)	0.37	−0.20± (−0.82,0.42)	0.53	−0.23± (−0.95,0.47)	0.51
	High risk	−0.45 (−1.06,0.14)	0.14	−0.63 (−1.28,0.01)	0.05	−0.22± (−0.84, 0.40)	0.48	−0.45± (−1.14,0.22)	0.19	−0.31± (−0.92,0.30)	0.31	−0.23± (−0.89,0.43)	0.49	−0.03± (−0.64,0.57)	0.90	−0.06± (−0.75,0.62)	0.85
TGy	Moderate risk	−0.83 (−1.57,−0.09)	0.02	−1.14 (−1.92,−0.37)	0.004	−0.40± (−1.12,0.30)	0.26	−0.47± (−1.24,0.29)	0.22	0.25± (−0.47,0.98)	0.49	0.27± (−0.50,1.05)	0.49	0.23± (−0.51.0.98)	0.54	0.05± (−0.78,0.89)	0.89
	High risk	−1.004 (−1.71,−0.28)	0.006	−1.38 (−2.12,−0.63)	< 0.001	−0.25± (−0.96,0.45)	0.48	−0.43± (−1.19,0.32)	0.26	0.26± (−0.44,0.97)	0.46	0.38± (−0.36,1.13)	0.31	0.26± (−0.45,0.99)	0.47	0.22± (−0.59,1.03)	0.59
Body composition																	
BRI	Moderate risk	0.68± (−0.58,1.94)	0.28	0.66± (−0.55,1.88)	0.28	0.03± (−1.25,1.32)	0.96	0.15± (−1.12,1.42)	0.81	−0.33± (−1.68,1.01)	0.62	−0.23± (−1.57,1.10)	0.72	0.31± (−0.98,1.61)	0.63	−0.06± (−1.37,1.24)	0.91
	High risk	−0.38± (−1.61,0.85)	0.54	−0.42± (−1.60,0.75)	0.48	−1.06± (−2.35,0.21)	0.10	−1.12± (−2.39,0.13)	0.08	−0.52± (−1.84,0.79)	0.43	−0.54± (−1.84,0.74)	0.40	0.52± (−0.72,1.78)	0.40	−0.02± (−1.28,1.23)	0.96
ABSI	Moderate risk	0.004± (−0.02,0.03)	0.76	0.01± (−0.01,0.04)	0.35	0.007± (−0.02,0.03)	0.63	0.009± (−0.02,0.03)	0.55	−0.03± (−0.06,−0.01)	0.008	−0.02± (−0.05,0.001)	0.05	−0.01± (−0.04,0.01)	0.27	−0.01± (−0.04,0.01)	0.26
	High risk	0.009± (−0.01,0.03)	0.48	0.01± (−0.01,0.04)	0.31	0.004± (−0.02,0.03)	0.78	0.003± (−0.02,0.03)	0.83	−0.02± (−0.05,0.003)	0.07	−0.02± (−0.05,0.005)	0.09	−0.003± (−0.02,0.02)	0.83	−0.007± (−0.03,0.02)	0.60
LAP	Moderate risk	−56.53± (−97.12,−15.94)	0.006	−78.06± (−120.23,−35.90)	< 0.001	−19.98± (−61.67,21.70)	0.34	−31.40± (−76.70,13.90)	0.17	−13.72± (−56.56,29.11)	0.53	−6.85± (−52.13,38.41)	0.76	−16.98± (−59.30,25.32)	0.43	−35.96± (−82.67,10.74)	0.13
	High risk	−66.72± (−105.95,−27.49)	0.001	−83.85± (−124.45,−43.25)	< 0.001	−23.61± (−64.98,17.76)	0.26	−35.75± (−80.72,9.21)	0.11	−8.72± (−50.26,32.81)	0.68	−2.36± (−46.32,41.60)	0.91	−3.04± (−44.08,38.006)	0.88	−15.79± (−61.17,29.59)	0.49
VAI	Moderate risk	−273.40± (−499.34,−47.45)	0.01	−362.91± (−611.44,−114.38)	0.004	−30.69± (−256.13,194.74)	0.79	−87.18± (−344.67,170.30)	0.50	−10.03± (−238.46,218.39)	0.93	31.88± (−221.62,285.40)	0.80	107.15± (−124.80,339.19)	0.36	109.72± (−162.67,382.12)	0.43
	High risk	−311.03± (−529.61,−92.45)	0.005	−393.001± (−632.49,−153.50)	0.001	−68.03± (−291.67,155.61)	0.55	−137.54± (−393.23,118.15)	0.29	−45.52± (−266.04,174.99)	0.68	−16.45± (−262.04,229.13)	0.89	118.24± (−106.92,343.42)	0.30	139.24± (−125.65,404.14)	0.30

## Discussion

The current cross-sectional study is a pioneering investigation aimed at establishing the interaction between the Genetic Risk Score (GRS) and plant-based diet on atherogenic factors and body fat distribution indices among women with overweight and obesity. Considering genetic susceptibility, we demonstrated that plant-based dietary patterns can influence cardiometabolic risk factors. Our results suggest that individuals with moderate or high genetic risk may benefit from plant-based dietary patterns in reducing cardiometabolic risk factors.

Our results revealed a significant negative interaction between tertile 2 of PDI and moderate risk allele and high-risk alleles, and AIP, TyG, LAP, and VAI compared to low-risk allele. Additionally, we observed a significant negative interaction between tertile 2 of PDI and high-risk allele with CRI. II compared to low-risk allele participants. Furthermore, there was a significant negative interaction between tertile 2 of hPDI and moderate risk allele and ABSI compared to low-risk allele participants. There was a positive significant interaction between tertile 2 of uPDI and moderate risk allele and CRI.I and ABSI compare to low-risk allele participants. The present study identified a significant mean difference between GRS and various anthropometric and metabolic indices, including body weight, BMI, WC, WHR, BRI, ABSI, and LAP.

Our results align with previous investigations regarding the genetic effects of GRS on BMI and waist circumference^74–76^. A cross-sectional study demonstrated the combined impact of specific genetic variants on obesity in Pakistanis, indicating the potential for predicting anthropometric traits using a GRS for obesity^77^. Several investigations indicated the positive associations between obesity risk factors such as BMI, WC, and WHR with cardiometabolic diseases^78–80^. CAV-1, one of the genes considered in this study, is a principal regulator of fat distribution and genetic lipodystrophy in humans. It can be more highly expressed in women with obesity compared to women who are thin^81–84^. CAV-1 is also associated with oxidative stress, which can play a role in many metabolic diseases. According to a study, following the PDI diet can reduce metabolic diseases among those with a risk allele in the CAV-1 gene^85^. Other roles, such as abnormalities in the binding of cholesterol and fatty acids, disturbance in the path of differentiation of fat cells, dysfunction of fat droplets, and increase in insulin signaling, are attributed to this gene^81,86^. In addition, several studies showed the association of the MC4R gene and its role in energy balance, food intake regulation, total fat, total obesity, peripheral obesity, abdominal obesity, and higher BMI^87,88^.

The results of the PREDIMED trial show that following plant-based dietary patterns can significantly reduce the risk of CVD^89^, and the Adventist Health Study also showed that vegetarian diets reduce CVD mortality by exerting their protective effect^90^. The PDI offers a comprehensive approach to evaluate various dietary indices, including the Alternate Healthy Eating Index, the Dietary Approaches to Stop Hypertension (DASH), and Mediterranean diet scores. This index incorporates multiple food items, encompassing healthy and less healthy plant-based foods, to effectively capture the diverse and combined intakes of various food sources. Moreover, some components in the uPDI, such as coffee, sugar-sweetened beverages, and saturated fat, could be involved in genetic predisposition to obesity^91–96^. Interactions between GRS and PDI food patterns may result from higher adherence to healthier plant-based foods and reduced consumption of unhealthy plant-based and animal foods, low energy density, the increased involvement of plant bioactive through the regulation of thermogenesis without energy consumption and shivering, as well as the improvement of the balance of intestinal microbiota due to the increase in dietary fiber consumption^96–99^. According to a study conducted in 2023 by Fatemeh Gholami et al. on 377 women with overweight and obesity, significant interactions between GRS and h-PDI were observed on body fat mass index, BMI, and waist circumference. In this study, the interaction between GRS and PDI was performed on some predictive factors of cardiovascular diseases, such as C-reactive protein, plasminogen activator inhibitor 1, and insulin. According to the findings of this study, following a plant-based food pattern despite the genetic differences in people seems to be a protective factor against the risks of cardiometabolic abnormalities^100^. According to past results, following more healthy plant-based diets in people with a higher genetic risk of obesity (regardless of basic obesity) causes more benefits in these people^101,102^. Our findings revealed a negative significant interaction between tertile 2 of PDI and moderate risk allele and high-risk allele and AIP compared to the low-risk allele. In a cross-sectional study conducted in 2023 by Farnaz Shahdadian et al., it was found that participants with the highest quartile of PDI, as well as the third quartile of hPDI, were associated with reduced odds of having high-risk AIP compared to the first quartile^103^. In this regard, other studies reported the relationship between PDI, especially hPDI, and their role in the management and prevention of high-risk AIP^104–106^. One of the reasons for the effect of a PDI on reducing high-risk AIP can be attributed to its role in lowering TG because AIP is a logarithm of the ratio of triglycerides and HDL^103^. Mahdieh Khodarahmi et al., in a cross-sectional study, observed that the interactions between the DASH score and MC4R rs17782313 genotypes on AIP among the female group were statistically significant^107^. An observational study found a significant interaction of GRS and hPDI on lipid factors. In this regard, other studies also reveal the beneficial effects of healthier plant-based diets in reducing TC levels and controlling HDL^101,108–110^. However, the results for TG have been inconsistent^109^.

Findings indicate a positive significant interaction between tertile 2 of uPDI and moderate risk allele with CRI.I compared low-risk allele participants and positive significant interaction between tertile 2 of uPDI and moderate risk allele with CRI.II compared to low-risk allele participants. In a study conducted in 2019 on 96 participants, CRI.I was considered as an index to predict cardiovascular risk among people in two diet groups: vegetarians and omnivores. In this study, CRI-I was significantly greater in omnivores than in vegetarians, and people who have higher adherence to a vegetarian diet elevate by 17 times the probability of having a normal CRI-I^111,112^.

Earlier studies have found the potential role of diet in body composition; however, little attention has been paid to LAP and TyG, which are strong indicators of cardiovascular disease^113,114^. We found a negative significant interaction between tertile 2 of PDI and moderate risk allele and high-risk allele with TyG compared to low-risk allele participants. Also, there was a negative significant interaction between tertile 2 of PDI and moderate risk allele and high-risk allele with LAP compared to low-risk allele participants. In a cross-sectional study conducted in 2020 by Mahshid Shahavandi et al. on 270 adults, the results indicated that more adherence to hPDI was associated with a decrease in BMI, WC, WHR, and LAP. However, in this study, there was no correlation between following PDIs and TyG^115^. In another study on Iranian adults in 2017, no significant relationship was found between healthy dietary patterns with the TyG index and visceral fat level^116^. However, a prospective cohort study conducted in 2020 found a negative association between an anti-inflammatory diet and the TyG index^117^.

Our findings showed a negative significant interaction between tertile 2 of PDI and moderate risk allele and high-risk allele with VAI compared to low-risk allele participants. Results show a negative significant interaction between tertile 2 of hPDI and moderate risk allele and ABSI compared to low-risk allele participants. Moreover, a positive significant interaction was demonstrated between tertile 2 of uPDI and moderate risk allele and ABSI. Also, there was a positive significant interaction between tertile 3 of uPDI and high-risk allele and ABSI compared to low-risk allele participants. Based on a cross-sectional study, a significant positive association was observed between fat intake and visceral adipose tissue (VAT) among overweight young adults. In this investigation, participants who followed a diet rich in carbohydrates, sugar, total fat, and saturated fat showed an increased mean in VAI and LAP. However, following a diet rich in vitamins, minerals, and fiber was associated with reduced levels of VAI and LAP^116^. In another study conducted in 2018 on older Americans, a negative association was indicated between the DASH diet index and VAI^118^.

### Strength and limitations

Our investigation is a novel study to evaluate the interaction of genetics risk score (GRS) and plant-based diet on atherogenic, visceral, and body adiposity. It examines certain factors that have not been considered in previous studies. The current study’s strengths include appropriate sample size and adjusting for potential confounders. However, there are some limitations, such as the design of the study, which cannot explain the causal relation between confounders. As our study was conducted on women who were overweight, our obtained findings cannot be generalized to other groups of society.

## Conclusion

The involvement of PDI, h-PDI, and uPDI appears to be a protective factor against cardiovascular risk factors in women with overweight and obesity with increased GRS. Prospective and interventional studies with greater sample sizes in different populations and ethnicities need to be conducted to further the knowledge about the interaction between PDI, hPDI, and uPDI with GRS, which are associated with atherogenic index and body composition.

## Data Availability

The datasets generated and/or analyzed during the current study are not publicly available [Due to ethics and related to the instructions issued by the fund source]. Still, they are available from the corresponding author on reasonable request. We state that all methods are based on the relevant guidelines and regulations.

## Abbreviations

GRS: Interaction of Genetics Risk Score
CVD: cardiovascular diseases
FFQ: food frequency questionnaire
PDI: plant-based diet index
hPDI: healthy plant-based diet index
uPDI: unhealthy plant-based diet index
GLM: generalized linear model
ABSI: A body shape index
BRI: body roundness index
WC: waist circumference
LAP: Lipid accumulation product
BAI: Body adiposity index
AIP: atherogenic index of plasma
CRI-I: Castelli risk index-I
CRI-II: Castelli risk index-II
TyG: triglyceride glucose
CAV-1: Caveolin-1
MC4R: melanocortin 4 receptor
Cry: Cryptochromes
NAFLD: non-alcoholic fatty liver disease
PCOS: polycystic ovary syndrome
FBS: Fasting blood sugar
GOD/PAP: Glucose Oxidase Phenol 4-Aminoantipyrine Peroxidase
T-Chol: total cholesterol
LDL: low-density lipoprotein
HDL: high-density lipoprotein
TG: triglyceride
FCT: Food Composition Table
ANOVA: Analysis of variance
